# Mothers and Fathers in NICU: The Impact of Preterm Birth on Parental Distress

**DOI:** 10.5964/ejop.v12i4.1093

**Published:** 2016-11-18

**Authors:** Chiara Ionio, Caterina Colombo, Valeria Brazzoduro, Eleonora Mascheroni, Emanuela Confalonieri, Francesca Castoldi, Gianluca Lista

**Affiliations:** a Centro di Ricerca sulle Dinamiche evolutive ed educative (CRIdee), Department of Psychology, Catholic University of Milan, Milan, Italy; b Neonatal Intensive Care Unit (NICU), V. Buzzi-Ospedale dei Bambini, ICP, Milan, Italy; VA Boston Healthcare System (Massachusetts Veterans Epidemiology Research and Information Center) Boston, MA, USA

**Keywords:** prematurity, NICU, parental stress, parenting, caring

## Abstract

Preterm birth is a stressful event for families. In particular, the unexpectedly early delivery may cause negative feelings in mothers and fathers. The aim of this study was to examine the relationship between preterm birth, parental stress and negative feelings, and the environmental setting of NICU. 21 mothers (age = 36.00 ± 6.85) and 19 fathers (age = 34.92 ± 4.58) of preterm infants (GA = 30.96 ± 2.97) and 20 mothers (age = 40.08 ± 4.76) and 20 fathers (age = 40.32 ± 6.77) of full-term infants (GA = 39.19 ± 1.42) were involved. All parents filled out the Parental Stressor Scale: Neonatal Intensive Care Unit, the Impact of Event Scale Revised, Profile of Mood States, the Multidimensional Scale of Perceived Social Support and the Post-Partum Bonding Questionnaire. Our data showed differences in emotional reactions between preterm and full-term parents. Results also revealed significant differences between mothers and fathers’ responses to preterm birth in terms of stress, negative feelings, and perceptions of social support. A correlation between negative conditions at birth (e.g., birth weight and Neonatal Intensive Care Unit stay) and higher scores in some scales of Impact of Event Scale Revised, Profile of Mood States and Post-Partum Bonding Questionnaire were found. Neonatal Intensive Care Unit may be a stressful place both for mothers and fathers. It might be useful to plan, as soon as possible, interventions to help parents through the experience of the premature birth of their child and to begin an immediately adaptive mode of care.

## Introduction

Preterm birth is defined by the WHO as birth that occurs before 37 weeks of gestation. Until the Nineties, prematurity was defined on the basis of birth weight, however, in recent years gestational age has been considered as the main indicator of physical and neurological maturation of preterm babies (Sansavini & Faldella, 2013). Preterm birth is a multi-problematic event that presents two main consequences: first of all, the medical and neurophysiological conditions of the new born baby put him or her in danger (particularly for infants with a weight lower than 1.500 grams and with a gestational age less than 32 weeks), and secondly, it could have a negative impact both on the mother and father’s relationship and on parent–child interactions (Müller-Nix & Ansermet, 2009). Although it has been widely demonstrated that preterm infants are at risk for developing deficit and delays, the underlying causes of these poorer developmental outcome, and the role of parents, are still less understood.

In particular, as far as we know still few studies investigate mothers’ and fathers’ initial experience and reactions immediately after the premature birth of their child (Aagaard & Hall, 2008; Arnold et al., 2013; Jackson, Ternestedt, & Schollin, 2003; Maroney, 1994; Orapiriyakul, Jirapaet, & Rodcumdee, 2007). Furthermore still few studies examined fathers’ experiences of preterm birth, although these studies highlighted the importance of fathers’ experiences (Candelori, Trumello, Babore, Keren, & Romanelli, 2015; Lundqvist & Jakobsson, 2003; Pohlman, 2005).

Preterm delivery potentially combines both biological and environmental risk factors, therefore, simple cause-and-effect models that identify preterm birth itself as the only cause for developmental disorder are lacking of predictive efficiency (Sameroff & Chandler, 1975). Sameroff and Chandler (1975) proposed a transactional model that described that children and parents influence each other. This model predicts that preterm birth itself does not cause negative developmental outcomes alone but that the stressful conditions following early delivery moderates the risk for later developmental difficulties.

One month after preterm birth, parents are shocked by the physiological and psychological conditions of their baby (Hoffenkamp et al., 2015; Singer et al., 2003). The event could interfere with their transitions into parenthood: the adverse medical condition of their baby prevents parents from taking care immediately of their new born child (Axelin, Lehtonen, Pelander, & Salanterä, 2010; Feldman, Weller, Leckman, Kuint, & Eidelman, 1999). When their baby stays in NICU^i^, parents usually feel powerless and helpless; therefore, they may be more stressed and vulnerable to emotional difficulties than parents of full-term babies (Clottey & Dillard, 2013; Sansavini & Faldella, 2013). Furthermore, preterm birth could be a traumatic event that affects parents’ everyday lives. In most cases premature birth is the unexpected result of medical complications for the mother, which makes necessary the immediate interruption of pregnancy, often in emergency situations, in order to avoid serious threats to the baby’s and mother’s health (Coppola & Cassibba, 2004). Referring also to the eight criteria for defining a potentially traumatic event, identified by Green (1990), premature birth could be a traumatic event since it is a threat to the physical integrity of the mother and a threat to the integrity of a loved one, the baby.

This trauma could lead parents to develop post-traumatic stress disorder symptoms of avoidance, hyperarousal, and intrusion (Lefkowitz, Baxt, & Evans, 2010), preventing parents from having a normative transition to parenthood (Watson, 2011) and damaging the new relationship between them and their baby (Ionio & Di Blasio, 2014).

Furthermore after birth the difficult medical conditions of premature babies and the mechanical environment in the NICU usually prevent a skin-to-skin relationship between children and their parents, and this could be dangerous for the future development of the babies (Wigert, Berg, & Hellström, 2010). Several studies have demonstrated the importance of parents’ extended presence and skin-to-skin contact with their infants for the infants’ long-term outcomes. Positive effects of skin-to-skin contact between children and their parents are related to effects on the parents (de Macedo, Cruvinel, Lukasova, & D’Antino, 2007; Feldman, Eidelman, Sirota, & Weller, 2002; Green & Phipps, 2015).

These findings are particularly important since a recent study pointed out that although the majority of units in different European countries reported a NICU policy that encourage both parents to take part in the care of their babies, parental involvement as well as the role played by mothers and fathers are generally more limited in Italy. Tasks involving more responsibility such as supporting the baby during uncomfortable procedures were commonly allowed in Sweden, United Kingdom and the Netherlands, but less in Italy. Furthermore in Italy many units applied restrictions regarding the frequency and the time of parents' admission to NICU and fathers are usually less involved than mothers as regards skin-to-skin contact and Kangaroo Care (Pallás-Alonso et al., 2012).

Parents of premature babies also have to leave their regular routines and spend many hours in the NICU, where they continue to experience the infants’ fragility and mortality (Clottey & Dillard, 2013). They live in a state of psychological and physical separation from their babies, aggravated by the artificial environment of the neonatal intensive care unit (NICU), in which the medical staff takes care of their infant’s neuropsychological and behavioral development and wellbeing, which often causes parents further pain and distress (Montirosso, Provenzi, Calciolari, Borgatti, & NEO-ACQUA Study Group, 2012; Sansavini & Faldella, 2013).

The sense of powerlessness and impairment could alter the parental role, and it could also increase anxiety, depression, helplessness, frustration, guilt, and anger (Müller-Nix & Ansermet, 2009).

In parents of preterm infants also external infant characteristics, associated with immaturity and severity of medical status, could be stressors that could further impair the very first relationship between parents and their baby (DeMier et al., 2000; Müller-Nix & Ansermet, 2009). The appearance of preterm babies is perceived as less physically attractive than the features of full-terms: they are immature and show less infantile facial features (Goldberg & DiVitto, 2002; Hildebrandt & Fitzgerald, 1979; Hoffenkamp et al., 2012).

In general parents should try to establish a balance between adaptive or non-adaptive behaviors in order to complete a functional transition to parenthood (Cena, Imbasciati, & Baldoni, 2010).

In order to make this transition, fathers and mothers have to perform different roles and develop specific competences. Different studies showed that after premature birth fathers usually have a supportive role. They are expected to help their wives, to contain their negative feelings such as sufferance, anxiety, and depression, and to think about both their wives and their babies, giving them proper support (Alkozei, McMahon, & Lahav, 2014; Lindberg, Axelsson, & Öhrling, 2007). Only after the discharge of their babies do fathers seem to be safer and more confident trying to establish a new active relationship with infants (Jackson et al., 2003). On the other hand, mothers try to establish a positive relationship with their newborn babies also by improving their abilities to take care of them and by learning new techniques from the medical staff. Sometimes, this type of interaction allows mothers to recover their self-esteem and their capacity to respond to their infants as an “expert” caregiver (Sansavini & Faldella, 2013). Furthermore only few studies on non-clinical sample have tried to identify the presence of significant differences between mother-child and father-child interaction. In particular some studies have shown that fathers are more prone to physical contact with their babies than mothers during the interaction (Lamb & Lewis, 2010). Furthermore this physical contact searched by fathers may affect the cognitive and socio-emotional child’s development both directly and indirectly, encouraging mother-child exchanges and supporting the triadic interactions within the family (Brown, McBride, Bost, & Shin, 2011).

Other studies have pointed out that parental stress experienced during the infant’s admission in NICU could influence the psychological and behavioral development of the baby (Dudek-Shriber, 2004; Howe, Sheu, Wang, & Hsu, 2014). Thinking about their babies as sick and in danger is very stressful for parents, and it may bring them to an emotional crisis (Alkozei et al., 2014; Franck, Cox, Allen, & Winter, 2005). For this reason, parents need to be informed about all the medical procedures carried out on their sick baby. Moreover, having a good relationship with medical staff and an active listener to their fears could reduce their anxiety (Müller-Nix & Ansermet, 2009).

Teti, Hess, and O’Connell (2005) reported that parents of premature babies are “preterm parents”: negative feelings, stress, anxiety, and the uncertain future of their babies put them in a position of fragility that could damage their attachment relationship with their babies (Coppola & Cassibba, 2004). Therefore, supporting parents during the hospitalization of infants could protect the development of the “preterm family” (Korja et al., 2010).

Since most of the studies examined focused on experiences over the longer period of the infant being hospitalized, they may analyze situations which have already become ‘normality’ for many parents. The main aim of the present study was to explore mothers’ and fathers’ experiences immediately after the premature birth of their babies and their first experience of NICU in order to increase our knowledge about mothers’ and fathers’ initial experience and reactions. In particular this study wanted to identify the most stressful factors for parents of premature babies and to specify the role of mother and father inside the NICU. In particular we wanted to investigate how mothers and fathers experience preterm birth immediately after the delivery and how they deal with the difficulties of this event and to understand how their feelings are linked to the NICU. Furthermore, we wanted to analyze the relation between the parents’ feelings and their premature children’s characteristics soon after their birth in order to better understand how this event influences the maternal and paternal response.

### Aim

Several studies (Matricardi, Agostino, Fedeli, & Montirosso, 2013; Miles, Holditch-Davis, Schwartz, & Scher, 2007; Sansavini & Faldella, 2013) have shown how premature birth (< 37 weeks) is often a traumatic event for parents that could cause in mothers and fathers trauma-related symptoms, such as symptoms of avoidance, intrusion and hyperarousal, negative states of mind and feelings, such as anxiety, hostility, inertia, depression, and bewilderment, and stress related to the NICU perception, the baby’s conditions and the perception of an altered parental role (Ionio & Di Blasio, 2014; Lefkowitz et al., 2010; Müller-Nix & Ansermet, 2009).

One of the aims of this study was to investigate maternal and paternal responses immediately after the premature birth of their child in terms of trauma-related symptoms, negative states of mind and feelings and stress related to the NICU perception, the baby conditions and the perception of an altered parental role by comparing a clinical sample of parents of preterm children and a control sample of parents of full-term children.

Furthermore, according to previous studies that showed that mothers experienced higher level of stress than fathers after their baby’s premature birth and NICU stay (Jackson et al., 2003), we wanted to better understand if mothers and fathers faced in a different way the premature birth of their babies in terms not only of trauma-related symptoms and negative states of mind and feelings but also of how they perceive the NICU, their baby conditions and their parental role and external support.

Finally, we wanted to understand how parental trauma-related symptoms, negative states of mind and feelings and stress related to the NICU perception, the baby conditions and the perception of an altered parental role in the preterm children sample were linked to the neonatal characteristic of the babies and to the hospitalization condition.

Based on previous studies (Ionio & Di Blasio, 2014; Lefkowitz et al., 2010; Matricardi et al., 2013; Miles et al., 2007; Müller-Nix & Ansermet, 2009), we expected:

1. the parents of preterm children would to show higher levels of trauma-related symptoms, negative states of mind and feelings and stress related to the NICU perception, the baby conditions and the perception of an altered parental role than parent of full-term children;
2. mothers to show more trauma-related symptoms, to feel more stressed, anxious, depressed and angry than the fathers and to respond more negatively to the situation, in particular as regards the NICU perception, the baby conditions and the perception of an altered parental role;
3. a correlation between higher levels of trauma-related symptoms, negative states of mind and feelings and stress related to the NICU perception, the baby conditions and the perception of an altered parental role in parents and the poor biological condition at birth, such as low gestational age and birth weight.

## Methods

### Participants

Twenty-one Italian couples (21 mothers and 19 fathers) of preterm infants and 29 Italian couples (29 mothers and 23 fathers) of full-term babies were contacted, who were recruited for a larger, longitudinal study that wanted to investigate the role of mothers and fathers on the neuropsychological development outcome of healthy preterm babies from birth until preschool age. The sample’s characteristics are summarized in Table 1.

**Table 1:** Demographic and Medical Characteristics of the Sample

Characteristics	Preterm (21 mothers and 19 fathers)	Full-Term (29 mothers and 23 fathers)	Differences
Demographic characteristics			
Maternal age (years)	*M* = 36.00, *SD* = 6.85	*M* = 34.92, *SD* = 4.56	*t*(34) = -1.35, *p* = .19
Paternal age (years)	*M =* 40.08, *SD =* 4.76	*M =* 40.32, *SD =* 6.77	*t*(32) = -.17, *p* = .87
Maternal education			χ^2^(2, *N* = 50) = 1.82, *p* = .40
<High School	0.0%	7.7%	
High School	18.2%	19.2%	
>High School	81.8%	73.1%	
Paternal education			χ^2^(2, *N* = 42) = 3.01, *p* = .22
<High School	25.0%	5.0%	
High School	25.0%	35.0%	
>High School	50.0%	60.0%	
Maternal profession			χ^2^(4, *N* = 50) = 3.78, *p* = .44
Elementary Occupation	4.6%	0.0%	
Craft Worker	0.0%	3.7%	
Service and Sale Worker	0.0%	3.7%	
Technicians	45.5%	55.5%	
Professional	49.9%	37.1%	
Paternal profession			χ^2^(5, *N* = 42) = 1.93, *p* = .86
Elementary Occupation	10.5%	11.2%	
Craft Worker	10.5%	7.1%	
Service and Sale Worker	26.4%	27.8%	
Technicians	0.0%	5.6%	
Professional	52.6%	42.7%	
Managers	0.0%	5.6%	
Infant medical characteristics			
GA (weeks)	*M* = 30.96, *SD* = 2.97	*M* = 39.19, *SD* = 1.42	*t*(36) = 13.13, *p* < .001
Birth Weight (g)	*M* = 1439.23, *SD* = 461.57	*M* = 3428.55, *SD* = 553.15	*t*(55) = 14.57, *p* < .001
Hospitalization (days)	*M* = 40.00, *SD* = 27.13	*M* = 3.87, *SD* = 0.85	*t*(25) = -6.79, *p* < .001

The clinical sample inclusion criteria were infant gestational age (GA) ≤ 37 weeks, absence of congenital anomalies.

Parents of preterm children were approached inside NICU, while parents of term children were approached inside the maternity ward by research psychologists, introduced by the medical doctors of the wards. After a brief meeting conversation, the research psychologists introduced the research to parents, explaining them that they would be involved in a longitudinal study in order to investigate preterm children development from birth till preschool age. If parents agreed to participate in the follow up study the researcher explained them the content of the questionnaire.

Approximately 50% of parents refused to participate in our research because they were totally focused on their child. Parents who agreed initially, usually did not change their mind, refusing to participate, except for two fathers who refused after reading the questionnaires, because they did not want to express their personal states and feelings.

### Measures

Parents filled out the following questionnaires; giving these questionnaires to parents, researchers specified that they have to complete them in regards to the recent birth of their child and their time spent in hospital:

**Impact of Event Scale Revised** (IES-R; Weiss & Marmar, 1997; Italian version by Pietrantonio, De Gennaro, Di Paolo, & Solano, 2003). This scale investigates trauma-related symptoms that characterize parents immediately after a potential traumatic event, in our case the premature birth of their child. It is composed of 22 items divided into 3 clusters rated on a 5 point Likert scale ranging from 0 (*not at all*), to 4 (*extremely*) that measure the presence of posttraumatic stress symptomatology: 8 items regarding symptoms of avoidance (parents’ avoidance of feelings, situations, memories; e.g. *“I tried not to think about it”*), 7 items regarding symptoms of intrusion (flashbacks, nightmares, images; e.g. *“I had dreams about it”*), and 7 items regarding symptoms of hyperarousal (fear, irritability, hypervigilance, and difficulties in concentration; *e.g. “I felt irritable and angry”*). There is also another scale that shows the total score obtained from the sum of others scales (avoidance, intrusion, hyperarousal). This scale showed good psychometric properties: internal consistency was Cronbach’s alpha = .96. For the current study Cronbach’s alpha reliability coefficients of mothers for the subscales were .85 (avoidance), .69 (intrusion), .86 (hyperarousal), and .66 for the total. Cronbach’s alpha reliability coefficients of fathers for the subscales were .61 (avoidance), .91 (intrusion), .77 (hyperarousal), and .60 for the total.

**Profile of Mood States** (POMS; McNair, Lorr, & Droppleman, 1971; Italian version by Farnè, Sebellico, Gnugnoli, & Corallo, 1991). This instrument investigates parents’ affective states and describes both negative and positive states of mind. It is used to define mothers’ and fathers’ profiles after preterm birth. It is composed of 58 items rated on a 5 point Likert scale ranging from 0 (*not at all*), to 4 (*extremely*) in order to examine 6 affective dimensions: tension–anxiety (e.g. “*Tense*”, “*Nervous*”), anger–hostility (e.g. “*Angry*”, “*Furious*”), fatigue–inertia (e.g. “*Fatigued*”, “*Exhausted*”), depression–dejection (e.g. “*Unhappy*”, “*Blue*”), vigor–activity (e.g. “*Active*”, “*Energetic*”), and confusion–bewilderment (e.g. “*Bewildered*”, “*Muddled*”). This measure showed excellent psychometric properties: internal consistency in the subscale ranged from Cronbach’s alpha = .63 (confusions) to Cronbach’s alpha = .96 (depression–dejection) (Curran, Andrykowski, & Studts, 1995). In the present study internal consistency range from Cronbach’s alpha = .69 (confusion–bewilderment) to Cronbach’s alpha = .93 (vigor–activity) for mothers and from Cronbach’s alpha = .70 (vigor–activity) to Cronbach’s alpha = .96 (depression–dejection) for fathers.

**Multidimensional Scale of Perceived Social Support** (MSPSS; Zimet, Dahlem, Zimet, & Farley, 1988; Italian version by Prezza & Principato, 2002). The MSPSS is a 12-item self-report questionnaire scored on a 7-point Likert scale ranging from 1 (*very strongly disagree*) to 7 (*very strongly agree*). There are three sub-scales addressing different areas of support, namely family (e.g. “*My family really tries to help me*”), friends (e.g. “*My friends really try to help me*”) and significant others (e.g. “*I have a special person who is a real source of comfort to me*”). This measured showed good psychometric properties: internal reliability is high (Cronbach’s alpha = 0.88). The authors report test-retest reliability of .85 over a 2-3 month period, along with moderate construct validity. For the current study Cronbach’s alpha reliability coefficients of mothers for the subscales were .92 (significant others) .98 (family) .98 (friends) and .96 for the total. Cronbach’s alpha reliability coefficients of fathers for the subscales were .81 (significant others) .80 (family) .97 (friends) and .87 for the total.

**Post-Partum Bonding Questionnaire** (PBQ; Brockington, Fraser, & Wilson, 2006; Italian translation by Montirosso, Fedeli, Provenzi, & Brockington, 2009). This questionnaire investigates possible difficulties and disorders in the relationship between the mother and father and their new born baby. The instrument is composed of 22 items rated on a 6 point Likert scale ranging from 0 (*always*), to 5 (*never*) divided into 4 subscales: the impaired bonding subscale (e.g. “*I feel happy when my baby smiles or laughs*”), the rejection and pathological anger subscale (e.g. “*I feel angry with my baby*”), the infant-focused anxiety subscale (e.g. “*My baby makes me feel anxious*”), and the incipient abuse subscale (e.g. “*I feel like hurting my baby*”). For the current study only impaired bonding subscale, rejection and pathological anger subscale and infant-focused anxiety subscale were used for analysis. Authors (Brockington et al., 2006) indicated that Scale 1 had a sensitivity of .82 for identifying all mother-infant relationship disorders; Scale 2 (rejection and pathological anger) had a sensitivity of .88 for identifying rejection of the infant, but only .67 for identifying severe anger. The performance of scale 3 (infant-focused anxiety) was unsatisfactory. Authors (Brockington et al., 2006) indicate that Scale 4 (incipient abuse) selected only a few mothers, but was of some value in identifying those at high risk of child abuse. For the current study Cronbach’s alpha reliability coefficients of mothers for the subscales were .62 (impaired bonding) .60 (rejection and pathological anger) and .60 for the total. Cronbach’s alpha reliability coefficients of fathers for the subscales were .70 (impaired bonding) .60 (rejection and pathological anger) .80 for the total. The performance of Scale 3 (infant-focused anxiety) both for mothers and for fathers was unsatisfactory as Brockington et al. (2006) version.

**Parental Stressor Scale: Neonatal Intensive Care Unit** (PSS:NICU; Miles, Funk, & Carlson, 1993; Italian version by Montirosso et al., 2012). This questionnaire was administered only to parents of preterm babies. This instrument describes parents’ stress and perceptions related to the NICU. This scale is composed of 34 items and is divided into 3 clusters: sights and sounds (of the machines of the NICU, e.g. “*The presence of constant noise of monitors and equipment*”), infant behavior and appearance (stress related to the infants’ status, e.g. “*The small size of my baby*”) and parental role alteration (stress related to the perceptions of parents’ strength to become a caregiver, e.g. “*Being separated from my baby*”). Parents had to rate each item on a 5-point Likert scale ranging from 1 (*not at all stressful*) 5 to (*extremely stressful*), The scale demonstrated high test–retest reliability, with correlations ranging from .69 for the subscales and .87 for the total scale. Internal consistency has also been demonstrated, with Cronbach’s alpha coefficients ranging from .73 to .92 for the subscales and from .89 to .94 for the total scale. In the current study this measure showed excellent psychometric properties with Cronbach’s alpha reliability coefficients of mothers and fathers ranging from .93 to .98.

### Procedure

Ethical approval was gained through the hospital’s research ethics committee, which required informed consent from each participant. So, informed consent for the protection of privacy (Law No. 196 of 2003) was a prerequisite to participating in the study. Parents both of preterm and full-term babies were recruited to complete all the questionnaires within 7–14 days from their infant’s birth. The parents were recruited from January 2013 to March 2014 in the neonatal intensive care unit and the maternal care unit of a hospital in Northern Italy.

### Analysis

Data were analyzed with SPSS Statistics Release 20.0. Since that the assumptions of parametric statistics were not satisfied (Shapiro Wilks test was significant for each variable) and because of the limited sample size the intergroup comparison between parents’ scores of preterm and full-term infants was done by an Independent Samples Mann-Whitney U non-parametric test. The comparison between mothers’ and fathers’ scores of preterm and full-term infants was done by a Related Samples Wilcoxon Signed Rank non-parametric test. Finally, correlations between parental questionnaire scores and gestational age, birth weight, and length of hospitalization in the preterm sample were done by the Pearson correlation test. The level of significance was set at *p* < .05.

## Results

### Parents of Preterm Children vs. Parents of Full-Term Children

Some significant differences in the POMS (McNair et al., 1971) scales were found (Table 2).

**Table 2:** Comparison of Preterm and Full-Term Mothers’ and Fathers’ Scores (POMS)

Line No.	Dimensions	Preterm		Full-Term		*p*
		*M*	*SD*	*M*	*SD*	
		Mothers				
1	Tension	53.76	11.675	45.97	8.542	.010
2	Depression	49.43	9.574	43.62	5.335	.000
3	Anger	51.57	13.094	43.48	6.332	.020
4	Vigor	50.19	11.707	62.76	10.456	.000
5	Fatigue	52.90	10.242	47.41	8.060	.030
		Fathers				
6	Anger	51.00	16.500	43.30	6.230	.004
7	Vigor	54.26	10.520	63.43	6.860	.008

Mothers of preterm babies showed higher scores than mothers of full-term babies in almost all the subscales of POMS (McNair et al., 1971), namely tension–anxiety (Line 1), depression (Line 2), anger–hostility (Line 3), and fatigue (Line 5). At the same time, these mothers appeared less strong than mothers of full-term babies: there is a significant difference between them in the vigor subscale (Line 4).

Fathers of preterm babies had a lower level of vigor–activity (Line 6) and a higher level of hostility–anger (Line 7) than full-term fathers had.

No significant differences were found in MSPSS (Zimet et al., 1988) and IES-R (Weiss & Marmar, 1997), even if mothers of preterm babies had generally higher scores.

### Mothers vs. Fathers in the Preterm Sample

No differences between mothers and fathers of preterm babies related to the POMS score (McNair et al., 1971) were found. Significant differences between mothers and fathers of preterm babies were found in the IES-R (Weiss & Marmar, 1997). Table 3 shows that mothers of preterm children had higher levels of intrusive feelings (Line 1), a higher tendency to be hyperactive (Line 2), and a significantly higher level of general stress (Line 3) than fathers of preterm babies.

**Table 3:** Comparison of the Scores of Mothers and Fathers of Preterm Babies

Line No.	Scales	Mother		Father		
		*M*	*SD*	*M*	*SD*	*p*
1	Intrusive Feelings (IES-R)	1.32	0.62	0.63	0.59	.00
2	Hyperarousal (IES-R)	0.90	0.79	0.46	0.77	.04
3	Total Stress (IES-R)	2.79	1.89	1.61	1.25	.01
4	Anger (PBQ)	0.80	1.37	1.92	2.21	.01
5	Total Score (PSS:NICU)	9.27	2.24	7.43	2.26	.01
6	Parental Role Alteration (PSS:NICU)	3.06	0.86	2.45	0.79	.02
7	Infant Behavior and Appearance (PSS:NICU)	3.73	0.94	2.76	1.28	.00

*Note.* IES-R scores are rated on a 5-point Likert scale (0 = not at all – 4 = extremely); PBQ scores are rated on a 6-point Likert scale (0 = always – 5 = never); PSS:NICU scores are rated on a 5-point Likert scale (1 = not at all stressful – 5 = extremely stressful).

Significant differences were found in the PBQ (Brockington et al., 2006): fathers showed significantly higher levels of anger than mothers (Line 4).

Some significant differences were found between mothers and fathers in PSS:NICU (Miles, Funk, & Carlson, 1993) scores: the alteration of parental role is higher in mothers than in fathers (Line 6), and mothers feel more stress related to the appearance of the babies than fathers do (Line 7) (Table 3).

Some significant correlations between the parental questionnaires scores of preterm children and gestational age, birth weight, and days of hospitalization were found in the preterm sample. Gestational age correlated negatively with anxiety and avoidance in fathers (IES-R) and with anger in mothers (PBQ). This means that the higher is the gestational age, the lower the stress and anger in parents will be. Birth weight correlated negatively with avoidance in fathers (IES-R) and anger in mothers (PBQ). The days of hospitalization correlated positively with mothers’ anger (PBQ), fathers’ avoidance, fathers’ total stress (IES-R), fathers’ depression, and fathers’ hostility (POMS). That means stress, anger, and negative feelings are higher when the baby spends more days in the NICU (Table 4).

**Table 4:** Correlation Among Parents’ Scores and Preterms’ Biological Condition at Birth in Preterm Children’s Sample

Variable	Maternal Anger (PBQ)	Paternal Avoidance (IES-R)	Paternal Anxiety (POMS)	Paternal Hostility (POMS)	Paternal General Stress (IES-R)	Paternal Depression (POMS)
Days in NICU	0.487*	0.565**	0.373*	0.397*	0.373*	0.414*
Gestational Age	-0.491*	-0.491*	-0.452*			
Birth Weight	-0.474*	-0.605**				

*p < 0.05. **p < 0.01.

## Discussion

Different studies widely demonstrated that preterm infants are at risk for developing deficit and delays, however the underlying causes of these poorer developmental outcome, and the role of the parent, are still less understood. In particular, since there is still a gap in literature about mothers’ and father’s initial experience and reactions to the premature birth of their child (Arnold et al., 2013), the main aim of the present study was to explore mothers’ and fathers’ experiences immediately after the premature birth of their babies and their first experience of NICU.

In order to respond to this main question, the first aim of this study was to investigate maternal and paternal responses immediately after premature birth of their child. Our results suggest that immediately after preterm birth parents of premature children experience the preterm birth as a stressful event that could increase stress and negative feelings such as anger, anxiety, and depression (Sansavini & Faldella, 2013). In particular our results pointed out that mothers of preterm children feel more anxious and more depressed than mothers of term children. Our results are in line with those researches that found that, at least one month after delivery, mothers of premature babies exhibited more negative feelings, in particular, anxiety and depression, than did the mothers of full-term children (Ravn et al., 2012; Vigod, Villegas, Dennis, & Ross, 2010).

Furthermore our results pointed out that in our sample both mothers and fathers of preterm babies were angrier than mothers and fathers of term babies. As Sansavini and Faldella (2013) showed, high levels of hostility, fear, and reactivity are dysfunctional to parenthood, and they may impair the relationship with the baby.

Finally, we found that after the birth of their child both mothers and fathers of preterm babies were more tired and less strong than mothers and fathers of full-term babies. Previous studies suggested that preterm birth usually causes negative thoughts in mothers and fathers: generally, they are not ready to deal with this event, and they may feel more weak and tired than parents of full-term children (Cena et al., 2010; Clottey & Dillard, 2013; Wigert et al., 2010). As these studies suggest, in our sample too mothers - mostly because of their physical conditions - and fathers exhibited higher levels of weakness and weariness. In fact, Wigert et al. (2010) highlighted that parents are tired after the birth of their baby, and some mothers needed care after a complicated and exhausting delivery. Parents needed to leave the NICU to recover themselves to be with their child.

A further aim of this study was to compare maternal and paternal experience and responses to the premature birth of their children. Our results, according to our initial hypothesis, show that immediately after the premature birth of their babies mothers had higher levels of stress and traumatic symptoms than fathers. Indeed previous studies about this topic showed that mothers, after their baby’s premature birth and NICU stay, felt more stressed than their husbands (Jackson et al., 2003) and sometimes, mothers could show significantly higher levels of post-traumatic stress symptoms such as avoidance, intrusion, and hyperarousal (Holditch-Davis, Bartlett, Blickman, & Miles, 2003).

On the other hand, fathers were angrier, more frightened, and less reactive than mothers (Lindberg et al., 2007). Even if there are still few studies that take into consideration the fathers’ prospective (Candelori et al., 2015; Lindberg et al., 2007; Lundqvist & Jakobsson, 2003; Pohlman, 2005), our results, according to these studies, added further evidence that underline that fathers need to be supported and understood (Lindberg et al., 2007). Indeed a metasynthesis of qualitative studies of fathers’ experiences identified themes of fear, frustration and issues of support (Steen, Downe, Bamford, & Edozien, 2012).

Furthermore, as regard how mothers and fathers experience the NICU environment our findings showed that mothers had higher levels of stress than fathers. This agrees with previous studies on mothers and fathers experience after preterm birth that pointed out that they participate in their infant’s care differently after birth (Pallás-Alonso et al., 2012; Trombini, Surcinelli, Piccioni, Alessandroni, & Faldella 2008). Mothers left their regular routines and spend many hours in the NICU, where they continue to experience the infants’ fragility and mortality (Clottey & Dillard, 2013). Furthermore they felt guiltier than fathers for the impairment of their premature child (Olshtain-Mann & Auslander, 2008). This could also infer that, as our results showed, mothers are more afraid than fathers about the behavior and appearance of their baby. Indeed, Olshtain-Mann and Auslander (2008) claim that mothers feel guiltier than fathers for their infant's prematurity. Furthermore, our results underline that the parental role is worse and more damaged in mothers than in fathers. In fact fathers usually are not expected to be actively involved in their infant’s care as mothers, and their participation helps them to cope with stress and empowers them in their paternal role (Lindberg & Öhrling, 2008). Our results therefore pointed out that fathers don’t experience the NICU as mothers do (Pallás-Alonso et al., 2012; Trombini et al., 2008).

Finally, the last aim of the present work was to understand how parental stress and negative feelings only in the preterm children sample were linked to the neonatal characteristic of the babies and to the hospitalization. We found that poor conditions at birth such as low gestational age, low birth weight and longer hospitalization are linked to higher levels of stress and more negative feelings such as anxiety, depression, and anger both in mothers and in fathers as literature suggests (Busse, Stromgren, Thorngate, & Thomas, 2013).

This study has also some limitations. First of all the cross-sectional data in this report cannot demonstrate how consistent these differences between the clinical and control samples are across time. A longitudinal design would allow a clearer vision of this. An additional benefit of a longitudinal design would be that it would allow an understanding of how differences between preterm and full-term parents are linked with their children’s development. Secondly our exploratory findings are taken from a single NICU and involved a limited sample size and his makes difficult to drawn strong conclusions. An important next step would be to include more participants in order to better understand our results. Including more participants could allow us to perform a regression model instead of correlations; indeed the correlational nature of our results prevents the examination of causality. A further limitation of the present study was that we included both first-time parents and parents that already have children and this may have an impact on the results since they could experience different levels of stress due to the novelty of the event birth. Including more participants could allow us to consider this variable as an independent variable that may have an impact on parental reactions after the preterm birth of their child. Finally the use of only self-report questionnaires to assess the constructs could be a further limit. It will be important to include new kinds of measures in the research design such as observational measure both of parents’ very first interaction with their babies and of parent’s interaction with the NICU environment and medical staff. Moreover, in order to better explain our results and go deeper in the parents’ experience it will be important consider qualitative measures as well, such as a semi structured interview addressed not only to parents but also to the medical staff.

### Conclusion

This study investigated mothers and fathers’ experiences immediately after the premature birth of their babies and their first experience of NICU.

Our findings have different implications. First of all our findings suggested that parents of premature babies, in particular mothers, since the birth of their babies, are at risk of developing higher levels of anxiety, depression, anger and stress. Furthermore, the preterm infants’ external characteristics and signals associated with immaturity and severity of medical status could be a further stressor especially for mothers. From previous studies (Diego, Field, Jones, & Hernandez-Reif, 2006; Feldman & Eidelman, 2007; Müller-Nix & Ansermet, 2009), we know that all these factors could have long-term effects on the quality of parent–infant interactions in the postnatal period. For these reasons our findings suggested that starting to support parents of preterm children from the very first moment after birth in order to reduce possible further negative consequences on preterm babies would be necessary.

Furthermore, considering both mothers’ and fathers’ point of view, we found that, even if in different ways, both parents are at risk after preterm birth. For this reason our findings suggested that a family-centered intervention is necessary in order to improve parents’ involvement in the care of their infant from the very first moment, to make them more conscious that they may have an active role in their infant’s development.
